# Generation of multimodal realistic computational phantoms as a test-bed for validating deep learning-based cross-modality synthesis techniques

**DOI:** 10.1007/s11517-025-03437-4

**Published:** 2025-09-27

**Authors:** Francesca Camagni, Anestis Nakas, Giovanni Parrella, Alessandro Vai, Silvia Molinelli, Viviana Vitolo, Amelia Barcellini, Agnieszka Chalaszczyk, Sara Imparato, Andrea Pella, Ester Orlandi, Guido Baroni, Marco Riboldi, Chiara Paganelli

**Affiliations:** 1 Department of Electronics, Information, and Bioengineering, Politecnico Di Milano, Milan, Italy; 2 Medical Physics Unit, Clinical Department, CNAO National Center of Oncological Hadrontherapy, Pavia, Italy; 3 Radiation Oncology Unit, Clinical Department, CNAO National Center of Oncological Hadrontherapy, Pavia, Italy; 4 Department of Internal Medicine and Therapeutics, University of Pavia, Pavia, Italy; 5 Bioengineering Unit, Clinical Department, CNAO National Center of Oncological Hadrontherapy, Pavia, Italy; 6 Department of Clinical, Surgical, Diagnostic,and Pediatric Sciences, University of Pavia, Pavia, Italy; 7 Department of Medical Physics, Ludwig-Maximilians-Universität München, Garching, Germany

**Keywords:** Imaging phantoms, Generative artificial intelligence, Radiotherapy planning

## Abstract

The validation of multimodal deep learning models for medical image translation is limited by the lack of high-quality, paired datasets. We propose a novel framework that leverages computational phantoms to generate realistic CT and MRI images, enabling reliable ground-truth datasets for robust validation of artificial intelligence (AI) methods that generate synthetic CT (sCT) from MRI, specifically for radiotherapy applications. Two CycleGANs (cycle-consistent generative adversarial networks) were trained to transfer the imaging style of real patients onto CT and MRI phantoms, producing synthetic data with realistic textures and continuous intensity distributions. These data were evaluated through paired assessments with original phantoms, unpaired comparisons with patient scans, and dosimetric analysis using patient-specific radiotherapy treatment plans. Additional external validation was performed on public CT datasets to assess the generalizability to unseen data. The resulting, paired CT/MRI phantoms were used to validate a GAN-based model for sCT generation from abdominal MRI in particle therapy, available in the literature. Results showed strong anatomical consistency with original phantoms, high histogram correlation with patient images (HistCC = 0.998 ± 0.001 for MRI, HistCC = 0.97 ± 0.04 for CT), and dosimetric accuracy comparable to real data. The novelty of this work lies in using generated phantoms as validation data for deep learning-based cross-modality synthesis techniques.

**Graphical Abstract:**

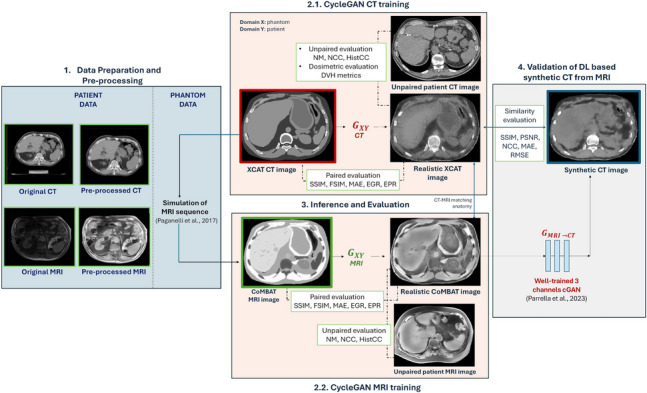

**Supplementary Information:**

The online version contains supplementary material available at 10.1007/s11517-025-03437-4.

## Introduction

In recent years, the use of artificial intelligence (AI) and its subfields, including machine learning and deep learning, has expanded across medical imaging, supporting tasks such as classification, segmentation, and image synthesis [1]. In this context, generative adversarial networks (GANs) have emerged as powerful tools for enhancing image quality [2, 3] and generating synthetic data [4–7].

Regarding the generation of synthetic data, GANs can be exploited to create realistic images to expand small datasets, which is particularly valuable in medical imaging settings where acquiring large, labeled datasets can be challenging. Several studies have demonstrated that synthetic images generated via GANs can effectively augment training datasets and improve model performance across a variety of tasks, from segmentation [4, 5] to lesion detection [6], and histopathology [7].

Another important application of GANs is cross-modality synthesis [8]. This application has recently gained relevance in external beam radiotherapy, where images can be transformed from one modality to another (e.g., converting MRI to CT), providing additional diagnostic information for dose calculation and treatment plan adaptation without the need for multiple scanning procedures [9]. In this scenario, paired acquisitions (i.e., where images from different modalities are perfectly aligned) are often necessary to train and test the model. However, due to set-up and anatomo-pathological variations between multimodal acquisitions, especially in thoraco-abdominal organs that move with respiration, these paired images are rarely available. Although several methods exist in the literature that allow for the use of unpaired data [10], thus mitigating the effects of organ motion between multimodal acquisitions during training, the scarcity of data and the absence of paired ground-truth volumes for validation purposes remain relevant obstacles, especially in some critical applications, such as for the generation of synthetic CT from MRI.

In this context, computational phantoms offer a potential solution by augmenting clinical datasets and providing ideal paired datasets unaffected by organ motion, making them highly useful for validation purposes [11].

However, incorporating computational phantoms into deep learning pipelines presents challenges because they do not fully represent the complexity of patient images and lack sufficient heterogeneity. Models trained solely on phantoms tend to generalize poorly when applied to actual patient data, whereas models trained exclusively on patient data cannot easily be validated using phantoms, due to the domain gap between the two. An example is the anthropomorphic computational XCAT phantom proposed by Segars et al. [12], which has been exploited for numerous applications [13–16] but lacks tissue heterogeneity within the organs. Moreover, the realism of medical images is not solely determined by anatomical heterogeneity, but also by modality-specific and scanner-specific acquisition characteristics. To bridge this gap, it is therefore necessary to enhance the realism of computational phantoms by transferring the style of patient images, including both anatomical and imaging features, onto the digital phantom anatomies.

This work aims to investigate the use of a CycleGAN for generating a library of patient-like computational CT and MRI phantoms and subsequently to exploit these paired realistic phantoms as a test-bed for validating deep-learning-based synthetic CT generation from MRI in the abdominal region for external beam radiotherapy. This approach offers a two-fold advantage: the generated realistic phantom dataset not only serves as an accurate motion-free ground-truth for validation but also extends the test dataset, addressing the limitation of data scarcity and enabling a more comprehensive evaluation of the model's performance.

## Related works

### Realistic XCAT phantoms

Different studies in the literature have exploited the XCAT phantom for validation purposes, also trying to replicate imaging protocols simulating CT, cone beam CT (CBCT), and MRI acquisition [17–20], but the lack of tissue heterogeneity in the phantom resulted in images that did not fully resemble real patient scans. Recently, several deep learning-based methods have been employed to enhance the realism of computational phantoms, demonstrating that synthetic data can effectively replicate key characteristics of patient data. In [21], the authors used a dual-discriminator conditional GAN to generate realistic synthetic XCAT phantoms, showing that radiomic features from synthetic phantoms followed the same distribution as those derived from patient CT images. In the work of Russ et al. [22], realistic CT images were generated from XCAT phantoms using an unpaired image-to-image approach based on CycleGAN. By combining both real and generated data, they were able to create a large, fully labeled dataset to enlarge the training dataset for a blood segmentation network. Furthermore, Al Khalil et al. [23] explored the effectiveness of realistic synthetic cardiac MRI images to boost heart cavity segmentation models by enlarging the training set. Starting from XCAT-phantom-based MRI simulations, a conditional image synthesis approach was used to replicate the style of patient images.

In the work of Bauer et al. [24], realistic CT, MRI, and CBCT images were generated from the XCAT phantom via CycleGANs, providing a robust tool to optimize registration parameters of a multimodal non-rigid registration technique.

Synthetic realistic data have predominantly been used for data augmentation [4–7, 22, 23]; however, the generation of realistic, perfectly aligned multimodal images from computational phantoms, can create reliable ground-truth datasets also for validation purposes. In [25], the importance of incorporating realistic phantom data into the validation process of AI models was emphasized. The authors incorporated realistic lung features into the ideal XCAT phantom and simulated CT and chest X-ray radiography (CXR) acquisition protocols to create synthetic COVID data for validating AI models trained on patient data to diagnose the infection using CT and CXR.

To the best of our knowledge, realistic computational phantoms derived via deep learning solutions have not yet been adopted for validating multimodal deep learning techniques for image synthesis tasks, such as for the validation of synthetic CT generated from MR images in external beam radiotherapy.

In Table 1, a summary of the different deep learning-based methods and applications for realistic XCAT phantoms is provided.

**Table 1:** Summary of studies using the XCAT phantom for realistic image generation

XCAT study	Modality	Method	Application
[21]	CT, CBCT	Dual-discriminator conditional GAN	Generation of realistic images
[22]	CT	CycleGAN	Generation of realistic images to augment training data for a blood vessel segmentation network
[23]	Cardiac MRI	Conditional GAN	Generation of realistic images to augment training data for a heart cavity segmentation network
[24]	CT, MRI, CBCT	CycleGAN	Multimodal non-rigid registration optimization
[25]	CT, CXR	Incorporation of morphological features in the XCAT and simulation of CT and CXR imaging protocols	Validation of AI models for diagnosis of COVID from CT and CXR images
**This study**	**CT, MRI**	**CycleGAN**	**Validation of a deep-learning approach for sCT synthesis from MRI for radiotherapy applications**

### Generation of synthetic CT in external beam radiotherapy

The generation of synthetic CT (sCT) from MRI images has gained interest in radiotherapy, as MRI offers high soft tissue contrast for precise delineation of the target volume and surrounding organs at risk in treatment planning. Additionally, its radiation-free nature allows for repeated acquisitions, potentially avoiding offline CTs for treatment adaptation that, instead, use X-rays and expose patients to additional, non-therapeutic radiation [26, 27].

Several works are present in the literature to derive synthetic CT from MRI data [28, 29], especially for the brain and pelvis regions which can be approximated as rigid bodies [30–33], whereas very few studies have addressed the thoraco-abdominal region [34–36] due to its anatomical complexity and presence of relevant organ motion introducing additional challenges.

A major challenge in cross-modality reconstruction stems from the fundamental differences in contrast mechanisms between CT and MRI. While CT captures electron density, MRI reflects tissue relaxation properties, resulting in a highly non-linear and complex mapping between the two modalities. This complexity is further compounded by modality-specific artifacts—such as susceptibility distortions in MRI—and differences in spatial resolution and field of view. Additionally, organ motion and inter-scan deformations, due to CT and MRI often being acquired at different times and under varying breathing conditions, further hinder accurate synthetic CT generation in the thoraco-abdominal site.

It should also be noted that, while synthetic CT has been widely explored for photon and proton therapies, applications in carbon ion radiotherapy (CIRT) remain limited. This is primarily because carbon-ion treatments are more sensitive to organ motion due to the sharper Bragg peak and increased biological effectiveness (RBE). In this context, organ motion can impact the accuracy of dose delivery.

Finally, a key limitation in sCT generation is the absence of ground truth data to evaluate the results of the deep learning approach. Indeed, to properly assess the performance of a deep learning network, the sCT needs to be compared with a reference CT perfectly matching the anatomy of the input MRI. The planning CT (or the CT closest in time to the MRI) is typically used to evaluate the performance, by registering the CT on the MRI through deformable image registration (DIR), although this procedure leads to sub-optimal results when CT and MRI show significantly different anatomy [27, 37–39].

In the context of MRI-only workflows in carbon-ions radiotherapy, the work of Parrella et al. [36] investigated for the first time the feasibility of a conditional GAN [40] (cGAN) in generating sCTs of the abdominal site. However, due to the lack of appropriate ground truth in patients’ data, a CT-MRI volume pair from the XCAT computational phantom was employed for validation purposes. Given that phantoms differ from patient data, a Gaussian noise filter was applied to blur the XCAT volume and simulate a more realistic ground-truth. While this first approach helped, there is the need for a more robust solution to align phantom data with the realism of patient imaging to fully exploit the advantage of digital phantoms in providing ground-truth for validating the performance of multimodal deep-learning based strategies.

### Rationale of the work

The key innovation of this work lies in leveraging realistically generated phantoms to validate AI methodologies, specifically for synthetic CT generation in radiotherapy applications. Traditional computational phantoms are inadequate for this purpose due to their limited and discrete intensity distributions, which introduce a substantial domain gap compared to clinical data typically used for training. Since GANs learn underlying data distributions, this mismatch results in artifacts, unrealistic outputs, and inflated error metrics.

In this study, the cGAN used for synthetic CT generation was trained on real patient data characterized by continuous, noisy, and heterogeneous features. When evaluated on clean, discretized phantom images, the model produced continuous-valued outputs (see Supplementary Fig. S1) that misalign with the discrete ground truth, artificially inflating error metrics (see Supplementary Table S1). This misalignment hinders meaningful model evaluation and risks misleading conclusions. Because GANs fundamentally operate as distribution learners, it is essential to validate them on data that closely matches the statistical properties of the training data distribution. Therefore, the main contributions of this study are as follows:

- Using CycleGAN networks to create a multimodal dataset from computational phantoms that statistically resemble clinical data while retaining known ground truth, addressing the challenge of paired multimodal dataset scarcity in medical imaging.
- Assessing the generated data through (i) paired and unpaired evaluations, along with a dedicated dosimetric analysis, ensuring clinical relevance in radiation therapy applications, and (ii) external validation on public CT datasets, to evaluate the generalizability of the approach.
- The use of generated phantom data to validate a synthetic CT generation model trained on real abdominal MRI, providing a controlled yet realistic test set for robust evaluation and bridging the gap between synthetic and real domains.

The organization of the remaining parts is as follows. Section 3 describes the materials and methods used in this study. It covers the phantom and patient datasets, the configuration of the CycleGAN model, and the evaluation pipeline. It also details the synthetic CT generation network that is being validated. Experimental results are presented in Section 4. In Section 5, a discussion is provided. Finally, conclusions are presented in Section 6.

## Materials and methods

### Overall workflow

**Fig. 1:**
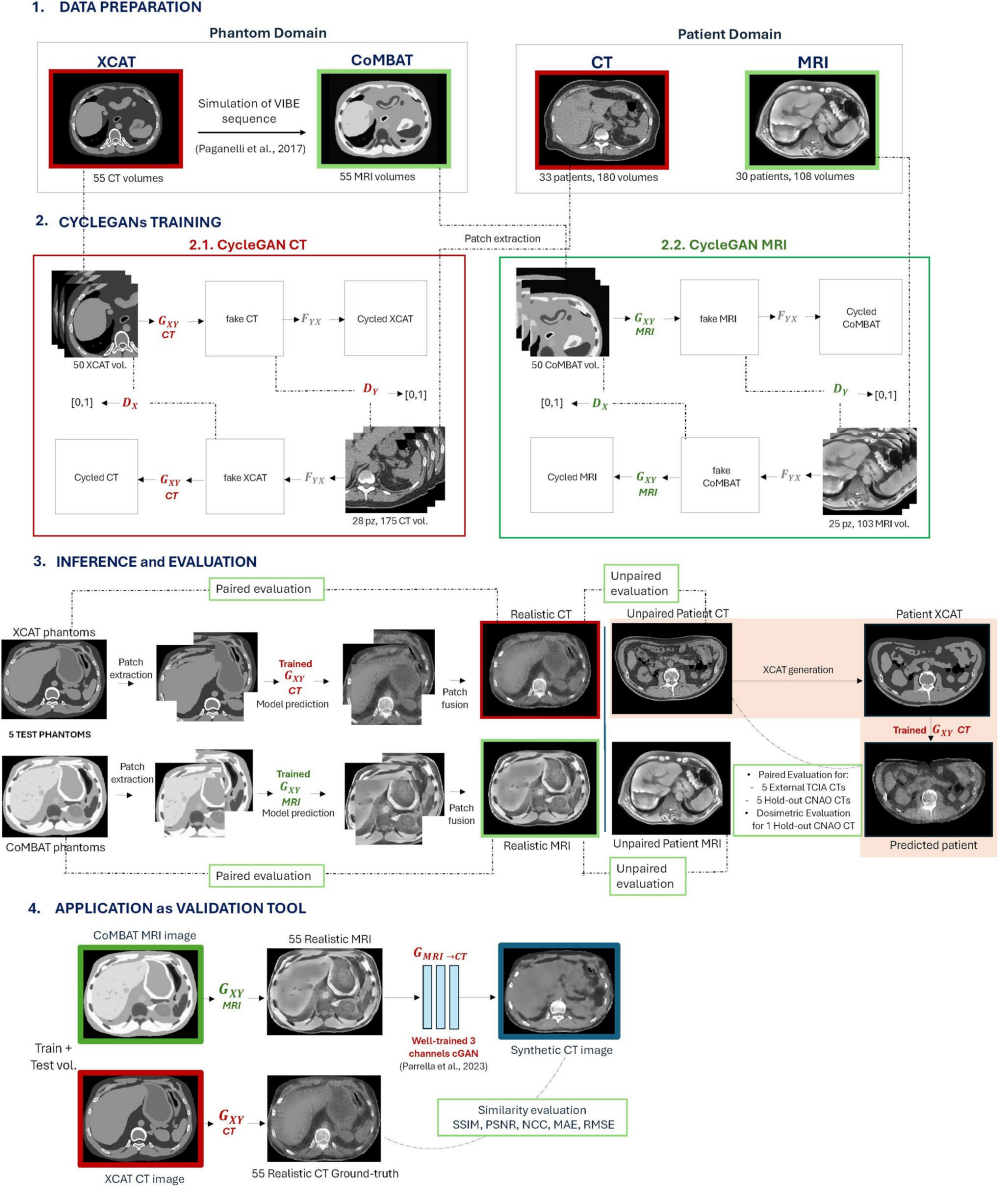
Overall workflow of the study. **A** Data preparation comprising the generation of XCAT computational phantoms and preprocessing of patient images. **B** Training of two independent CycleGANs, one for each imaging modality. **C** Patch-based prediction on test phantoms to evaluate the performance of the networks in style transfer. **D** validation of a GAN from the literature for sCT generation using realistic data

The proposed work consists of the following four steps, illustrated in Fig. 1.

1. The first step involves the construction and preparation of a multimodal dataset (i.e., CT and MRI) that includes abdominal images from both patients and XCAT phantoms.
2. The second step focuses on training two CycleGAN networks (one for CT and one for MRI), designed to transfer the style of patient images onto the anatomies available in the XCAT library. The network training was conducted on a patch basis.
3. To quantitatively evaluate the results, the generated volumes (i.e., realistic phantoms) were assessed from multiple perspectives: (i) paired evaluation, which quantifies the anatomical accuracy relative to the original phantom, (ii) unpaired evaluation, which measures realism compared to patient data, and (iii) patient-specific phantom generation and dosimetric evaluation, which aims to demonstrate the clinical realism of the predicted volumes by comparing a CIRT plan calculated on a patient CT with the same plan recalculated on the corresponding realistic CT derived from a patient-specific XCAT phantom. In addition, an external validation was performed, relying on independent CT data from an open-access repository.
4. Finally, the realistic, paired CT/MRI phantoms were used as an augmented test-bed to validate a deep-learning model from the literature that generates abdominal sCT images from MRI data for applications in CIRT.

### Datasets and data preparation

#### Patient data

The patient dataset consisted of 26 cases diagnosed with abdominal (pancreatic/liver) cancer and treated with CIRT at the National Centre for Oncological Hadron therapy (CNAO, Pavia). For each patient, a 4DCT, a 3D breath-hold T1-weighted MRI, and a free-breathing 4D T2-weighted MRI were acquired on the same day. The 4DCT provided a volume for each of the four pre-defined respiratory phases: end-exhale (0EX), 30%-exhale (30EX), 30%-inhale (30IN), and 100%-inhale (100IN). For some patients, additional respiratory-binned volumes were derived (up to 8). The 3D T1-w MRI was acquired at the end-exhale phase with the VIBE sequence (volumetric interpolated breath-hold examination). The free-breathing 4D T2-weighted MRI was derived via retrospective sorting with a limited field of view, and the quantified respiratory motion was used to deform the 3D T1-w MRI to derive a full-FOV virtual 4D T1-w MRI, as described in the work of Meschini et al. [13]. For some patients, re-evaluation CT and MRI scans were acquired during the treatment course, and these were considered independent of the first acquisition. All volumes from the different respiratory phases were included in the patient dataset to increase the number of available images, resulting in 180 abdominal CT volumes and 108 MRI volumes.

CT volume acquisitions had an in-plane size of 512 × 512 pixels, with the number of slices ranging from 96 to 145 and a voxel spacing of 0.98 × 0.98 × 2 mm^3^, while T1-w MRI acquisitions were acquired with a resolution of 1.0625 × 1.0625 × 3 mm^3^ (repetition time TR = 3.87 ms, echo time TE = 1.92 ms, flip angle *α* = 9°), an image size of 320 × 260 pixels, and 64 transversal slices.

Both patient CTs and MRIs underwent preprocessing to improve image quality. CTs were clipped between − 1000 HU and + 1047 HU, and the background was set to − 1000 HU using a body-mask built by CT thresholding and automatic refinement. More in detail, the mask was generated by applying thresholding at − 110 HU, followed by hole filling, edge refinement using a Sobel filter, and largest contour extraction to isolate the body region. They were also resampled to the corresponding MRI voxel spacing of 1.0625 × 1.0625 mm^2^ on the axial plane, resulting in an image size of 470 × 470 pixels. Patient MRIs were preprocessed as described in the work of Parrella et al. [36].

Five patients were selected as the test set for each imaging modality, with the remaining volumes forming the training set. For each test patient, only the volume at the 0EX respiratory phase was included in the test set; all other respiratory phases were excluded from the study at all stages, ensuring no overlap with training data.

#### Phantom data

The phantom dataset was created using the 4D Extended Cardiac-Torso (XCAT) Phantom [12], a parameterized digital model that provides highly detailed body anatomies through non-uniform rational B-splines (NURBS). Fifty-five different XCAT computational phantoms were produced using the complete library of adult anatomical models available. For each phantom, the corresponding attenuation map, providing the linear attenuation coefficient *µ* for each tissue, was generated at an energy level of 120 keV and subsequently converted into CT numbers.

The original phantoms had a volume size of 370 × 370 × [700–1200] voxels and a voxel spacing of 1 × 1 × 1 mm^3^. The abdominal region only was maintained, then they were resampled to the voxel spacing of the patient data (1.0625 × 1.0625 × 2 mm^3^), leading to an image size of 348 × 348 pixels in the axial plane.

Starting from the CT XCAT phantom, the corresponding MRI phantom versions were generated by simulating the VIBE sequence with CoMBAT [20].

Fifty CT XCAT phantoms and their corresponding MRI volumes composed the training set, while five CT-MRI phantoms were used as the test set for the evaluation step.

#### Data preparation

XCAT CT phantoms and unpaired patient CT images were used to train the CycleGAN for CT, whereas MRI phantoms and unpaired patient MRI images were used to train the CycleGAN for MRI. Both datasets were constructed following identical steps: (i) a linear intensity transformation to rescale the original intensity range to [− 1; + 1], (ii) extraction of 2D patches from each axial slice, and (iii) resizing of the patches to 256 × 256 pixels to match the input size required by the network architecture.

Due to different image dimensions across MRI, CT, and phantom domains, patches were extracted from each axial slice using different patch sizes and stride values. Specifically, patches of size 200 × 200 pixels were extracted from MRI images with a stride of 32, while patches of dimensions 220 × 220 pixels were extracted from CT and phantom images using a stride of 32 and 64, respectively.

These parameters were defined to minimize the extraction of patches with overly similar information content, while also avoiding patches with excessive background.

Then, 4 patches were randomly selected from the extracted ones, obtaining multiple patches per axial slice instead of just one. We chose to work with patches both to augment the data for training and to focus on local image details, which is crucial for achieving high-resolution synthetic images.

### CycleGANs training

The CycleGAN architecture was adapted from the one proposed by Zhu et al. [10], which enables image-to-image translation using unpaired training data. It consists of two generators—*G* and *F*—and two discriminators—*Dx* and *Dy*—which learn two mappings: a forward mapping from domain *X* to domain *Y* (via *G*), and a backward mapping from *Y* to *X* (via *F*). Generator* G* converts images from domain *X* (e.g., phantom) into domain *Y* (e.g., patient), while *F* performs the inverse translation. The discriminators *Dx* and *Dy* evaluate the realism of the generated images in both domains. The adversarial training process drives the generators to progressively improve their ability to produce realistic images, while the discriminators simultaneously become better at distinguishing generated from real images. Throughout training, this competition brings the system to an equilibrium where the generators produce images that are indistinguishable from real ones, and the discriminators can no longer differentiate between real and generated images.

**Fig. 2:**
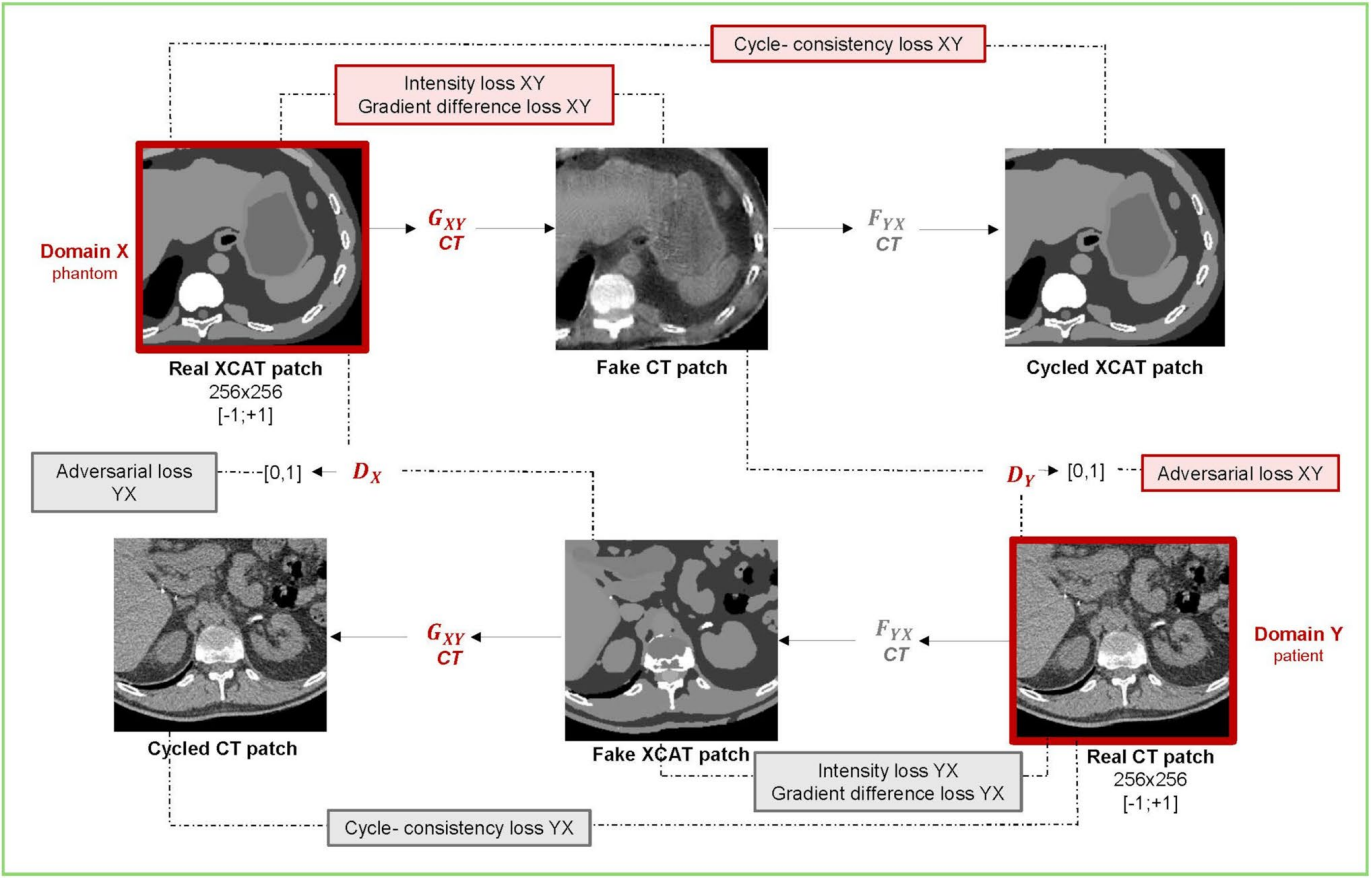
CycleGAN for CT images: generator G_XY_ aims to translate an XCAT patch to a CT patch. Discriminator D_Y_ aims to distinguish between patient CTs and those predicted by G_XY_. Vice versa for generator F_YX_ and D_X_. Analogously applies for CycleGAN MRI

In this study, generator *G* was used to translate phantom images into realistic patient-like images, while generator *F* translated patient images into phantom-like images. Figure 2 illustrates the CycleGAN architecture for CT, with an analogous scheme used for MRI.

#### Network architecture

**Fig. 3:**
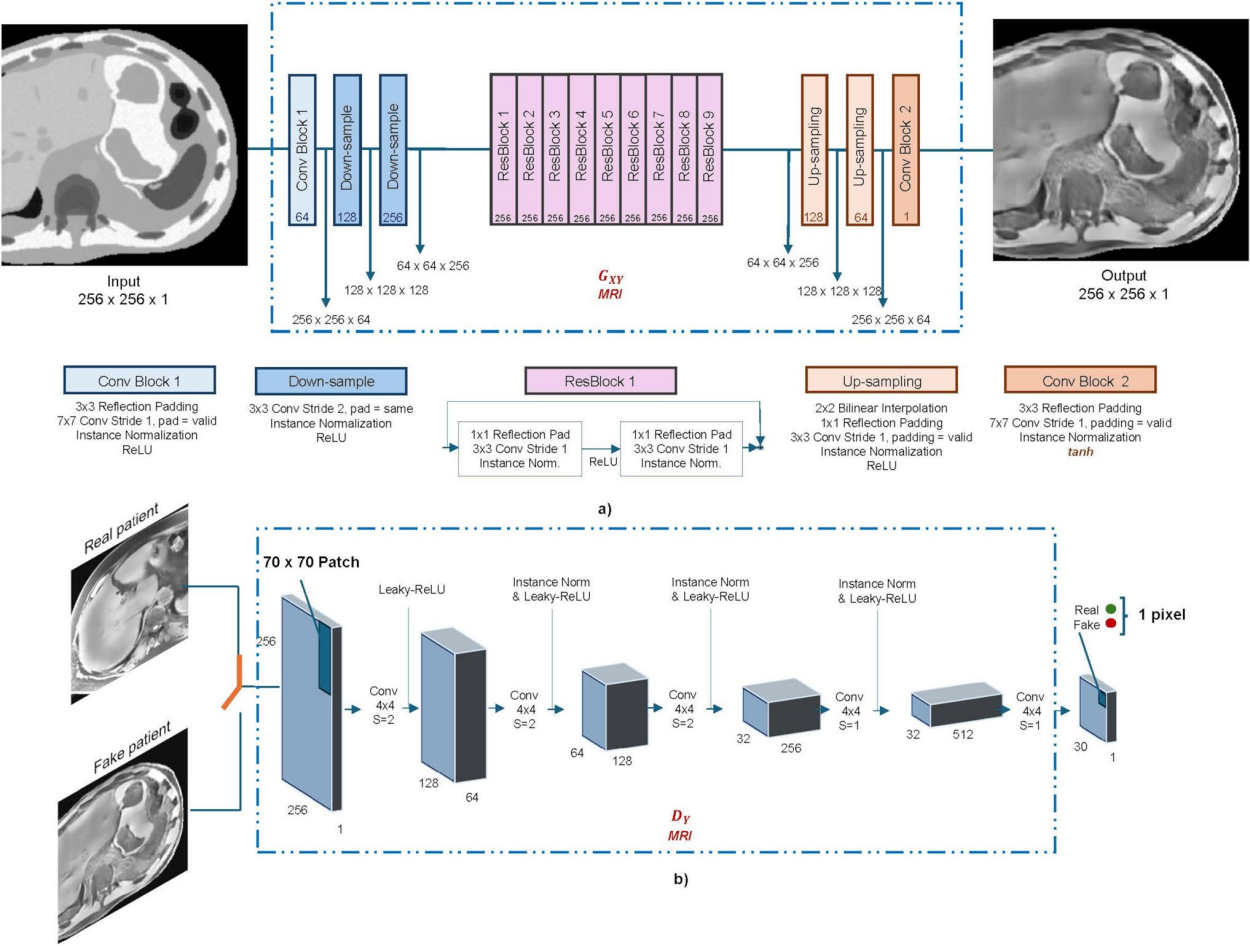
Network architecture. **a** ResNet architecture for both generators of CycleGANs. The key architectural details include the use of reflection padding to reduce boundary artifacts, instance normalization, and bilinear upsampling to avoid checkerboard artifacts in the generated image. In total, 9 residual blocks were employed for Handling inputs of size 256× 256 pixels. **b** 70 × 70 PatchGAN architecture for discriminators. It processes one input image at a time and outputs a matrix with each pixel classifying a 70 × 70 patch of the input. A pixel value of 1 indicates the patch is from the real domain, while 0 indicates it is from the fake domain

The ResNet configuration was used for the two generators of both CycleGAN networks [22]. It consisted of three encoding blocks for downsampling the input image, followed by nine residual blocks, and two upsampling layers for decoding. Upsampling based on bilinear interpolation was used to prevent checkerboard artifacts in the generated images. The discriminators employed a 70 × 70 PatchGAN architecture. For a 256 × 256 pixels input image, the discriminator returned a 30 × 30 output map, where each pixel classified a 70 × 70 input patch. The input images were downsampled through 5 convolutional layers. A schematic illustration of the architecture of generators and discriminators is depicted in Fig. 3.

#### Loss functions

During the training of the CycleGANs, each of the four architectures was optimized independently at each iteration. For CycleGAN CT, the loss function for generator ${G}_{XY}$ in Fig. 2 (responsible for translating from *X,* the phantom, to *Y,* the patient CT) was trained by minimizing a loss function defined as the weighted sum of several terms:

1 $$L\left({G}_{\mathrm{XY} }\right)= {L}_{\mathrm{GAN}}\left(\mathrm{XY}\right)+ {\lambda }_{cyc}\left({L}_{cyc}\left(\mathrm{XY}\right)+ {L}_{cyc}\left(\mathrm{YX}\right)\right)+ {\lambda }_{int}{L}_{int}(\mathrm{XY})+ {\lambda }_{gdl}{L}_{gdl}\left(\mathrm{XY}\right)$$

with ${L}_{\mathrm{GAN}}$ the generative adversarial loss, ${L}_{cyc}$ the cycle-consistency loss, ${L}_{int}$ the intensity loss and ${L}_{gdl}$ the gradient difference loss [41]. The coefficients *λ*_cyc_/*λ*_int_/*λ*_gdl_ control the relative weight of the different loss terms.

The adversarial loss is central to the training process, since it guides the optimization of the generators through the discriminator output, i.e., it drives the generator to create images that look realistic to the discriminator. In the case of CycleGAN CT, the generator ${G}_{XY}$, responsible for generating realistic CT images (domain *Y*), uses the feedback from the discriminator ${D}_{Y}$ to learn how to refine the generation process to make it more indistinguishable from patient CT scans. The adversarial loss, based on binary cross-entropy (BCE), is calculated as

2 $${L}_{\mathrm{GAN}}\left(\mathrm{XY}\right)=-\mathrm{log}\left({D}_{Y}\left({G}_{\mathrm{XY}}\left(X\right)\right)\right)$$

where ${G}_{XY}\left(X\right)$ is the generated realistic CT image from the input phantom *X*, and ${D}_{Y}$ is the discriminator that outputs a probability that the image is real or fake. As the generated image approaches realism, ${D}_{Y}$’s output approaches 1, thus minimizing the adversarial loss.

Because the mapping between domains *X* and *Y* is ambiguous, with multiple valid translations possible, CycleGAN incorporates a cycle-consistency loss: by mapping an image from *X* to *Y* and then back from *Y* to *X*, the original input should be recovered. It uses the image generated by the first generator as the input to the second generator and measures the difference between the real image and the cycled-reconstructed one using the L1 norm. The cycle-consistency loss is computed as the sum of the forward loss ${L}_{\mathrm{cyc}}\left(\mathrm{XY}\right)$ (computed between the original phantom image and its cycled reconstruction) and the backward loss ${L}_{\mathrm{cyc}}\left(\mathrm{YX}\right)$ (computed between the original patient image and its cycled reconstruction), since the original image and the reconstructed cycled image involve both generators *G* and *F*. Therefore, ${L}_{\mathrm{cyc}}\left(\mathrm{XY}\right)+ {L}_{\mathrm{cyc}}(\mathrm{YX})$ enforces that translation in both directions is consistent.

Additionally, an intensity loss and a gradient difference loss were incorporated into the generator loss, as proposed by Russ et al. [22] and Bauer et al. [24]. For ${G}_{XY}$, both losses are computed between the input phantom image X and the corresponding generated realistic image ${G}_{XY}\left(X\right)$. The intensity loss, calculated using the L1 norm, aims to preserve the pixel intensities of the organs within the phantom:

3 $${L}_{\mathrm{int}}\left(\mathrm{XY}\right)=\Vert {G}_{XY}\left(X\right)-X\Vert.$$

The gradient difference [42] loss ensures that edges and sharp boundaries between different tissues or anatomical structures are preserved during translation. It compares the first-order gradients (i.e., pixel-to-pixel differences) between the input image *X* and the generated image $Y= {G}_{XY}\left(X\right)$, penalizing discrepancies in gradient behavior along both the horizontal (*x*-axis) and vertical (*y*-axis) directions. Mathematically, it is computed as

4 $${L}_{\mathrm{gdl}}\left(\mathrm{XY}\right)= \sum_{i,j}{\left|\left.\left|\left.{X}_{i,j}-{X}_{i-1,j}\right|\right.-\left|\left.{Y}_{i,j}-{Y}_{i-1,j}\right|\right.\right|\right.}^{2}+ {\left|\left.\left|\left.{X}_{i,j}-{X}_{i,j-1}\right|- \left|\left.{Y}_{i,j}-{Y}_{i,j-1}\right|\right.\right.\right|\right.}^{2}$$

where ${X}_{i,j}$ and ${Y}_{i,j}$ are the pixel intensities at location (*i,j)* in the input and generated images, respectively. ${X}_{i,j}-{X}_{i-1,j}$ is the vertical gradient, while ${X}_{i,j}-{X}_{i,j-1}$ is the horizontal gradient.

Discriminators are trained to correctly classify the real and generated images by means of the BCE. The total loss for ${D}_{Y}$ is penalized when ${D}_{Y}$ fails to classify a real image correctly and when it incorrectly classifies a generated image. It is defined as the combination of two terms:

5 $$L\left({D}_{Y}\right)=\frac{1}{2}\left[{\mathrm{BCE}(1,D}_{Y}\left(Y\right))+ {\mathrm{BCE}(0, D}_{Y}\left({G}_{\mathrm{XY}}(X)\right))\right]$$

where ${D}_{Y}\left(Y\right)$ represents the discriminator’s output when evaluating a real image *Y*, while ${D}_{Y}\left({G}_{XY}(X)\right)$ is its output for a generated image. ${D}_{Y}$ is trained to output 1 when it classifies real images and 0 when it classifies generated images. Dividing the discriminator loss by a fixed factor helps balance the learning dynamics between the generator and the discriminator, preventing one from overpowering the other during training. Following the original CycleGAN implementation by Zhu et al. [10], we adopted the same fixed factor of 2. This choice was supported by preliminary experiments, which showed stable training behavior.

#### Training details

Most of the training configurations were adopted from the original study [10]. To stabilize training, the discriminators were updated using a randomly selected image from a buffer of K images (with K = 50), rather than using the most recently generated image. Both CycleGAN CT and CycleGAN MRI were trained with a batch size of 1 for 150 epochs; the Adam optimizer was used to minimize the loss functions of both generators and discriminators.

We performed a dedicated hyperparameter tuning process for both networks. Specifically, we optimized the weighting of the additional loss terms—intensity and gradient difference loss—as well as the learning rate of the discriminator. In this process, the cycle-consistency loss weight was fixed at 10, the adversarial loss weight at 1, and the generator learning rate at 0.0002. These fixed values correspond to the standard settings used in the original CycleGAN paper [10]. The best epoch for each configuration of hyperparameters was selected through visual inspection of the generated test volumes.

Given the limited patient dataset size, we checked the risk of overfitting by employing data augmentation based on patch extraction to increase the diversity of training samples. To ensure that the model generalized well to unseen cases, we also performed external validation using public CT datasets and compared the results with holdout cases representing the clinical scenario of interest as seen during training (see Sections 3.4.3 and 3.4.4).

### Inference and evaluation

Five XCAT phantoms and their corresponding MRI phantoms were used to quantitatively evaluate the performance of the two networks in transferring realistic patterns onto the original phantom anatomy. The inference process was conducted on a patch basis: each axial slice, sized 348 × 348 pixels, was divided into 9 patches of dimensions 220 × 220 pixels according to the training procedure and normalized between [− 1; + 1]. Each patch was then resized to 256 × 256 pixels for model prediction, mapped back to the patient range of values [− 1000; + 1047], and the final realistic image was obtained through a mean patch fusion technique, where overlapping patches were averaged to ensure smooth transitions and reduce boundary artifacts in the final image.

The generated realistic CT and MRI volumes were assessed using (i) a paired evaluation with the corresponding original phantoms, (ii) an unpaired evaluation with unmatched patient scans, and (iii) a dosimetric evaluation with a patient-specific ground truth. Then, we conducted an independent validation study by applying the trained CycleGAN CT to synthetic data (i.e., XCAT phantoms) derived from public CT datasets. Finally, to further explore the applicability of the realistic CT/MRI phantoms, we employed them as ground-truth data to test a deep learning method from the literature for abdominal synthetic CT generation from MRI, as proposed by Parrella et al. [36].

#### Paired evaluation

To evaluate the anatomical accuracy of the generated realistic CT and MRI volumes, axial slices were compared to the corresponding slices from XCAT and CoMBAT phantoms using several metrics: mean absolute error (MAE), structural similarity index (SSIM) [43], feature similarity index (FSIM) [44], edge preservation ratio (EPR) [45], edge generation ratio (EGR) [22], and the learned perceptual image patch similarity (LPIPS) metric [46]. MAE measured voxel-wise intensity differences to quantify changes from the original phantom.

SSIM and FSIM assessed the similarity of structural and feature details, respectively, while EPR and EGR focused on image sharpness and edges. To compute EPR and EGR, binary edge maps (EMs) were first extracted from both realistic and original phantom images using the Canny edge detector. Mathematically, the metrics were defined as follows:

6 $$\mathrm{EGR}= \frac{\mathrm{num}({\mathrm{EM}}_{\mathrm{generated}})}{\mathrm{num}({\mathrm{EM}}_{\mathrm{original}})}$$

7 $$\mathrm{EPR}= \frac{\mathrm{num}({\mathrm{EM}}_{\text{original }}\cap {\mathrm{EM}}_{\mathrm{generated}})}{\mathrm{num}({\mathrm{EM}}_{\mathrm{original}})}$$

where $\mathrm{num}$ represents the number of pixels. EGR measures the ratio of edge pixels in the generated image to those in the original image, reflecting the amount of edge generation. EPR measures the ratio of correctly preserved edge pixels between the original and generated images relative to the original, indicating edge preservation performance. High EPR values indicate strong edge preservation, while EGR values close to 1 reflect a minimal generation of spurious edges.

LPIPS was additionally included to assess perceptual similarity from a deep learning perspective. For each pair of corresponding XCAT and realistic images, a 256 × 256 patch was extracted from the center at the same spatial position in both images. These corresponding patches were then used to compute LPIPS, providing a feature-based comparison that reflects human perceptual similarity more closely than pixel-wise metrics. For LPIPS, lower values (closer to zero) indicate greater perceptual similarity.

Because the network modifies the original phantom to generate a more realistic image, a perfect SSIM (i.e., 1.0) is not expected. Instead, metrics are used to confirm that structural, perceptual, and edge-level characteristics remain consistent with the original phantom without excessive distortion. Values of SSIM and FSIM above 0.5 indicate that critical structures and textures are retained. Similarly, LPIPS values were used to verify that perceptual differences remained within an acceptable range. Meanwhile, edge generation ratio and edge preservation ratio are employed to confirm that the anatomical structures of the phantom are retained and not overly distorted.

Paired metrics were computed separately for CT and MRI datasets on 2262 axial slices across the five test phantoms. The calculations were performed for each slice pair and averaged over the entire set of slices.

#### Unpaired evaluation

To assess the realism of the generated CT and MRI volumes from CycleGANs, the predicted 3D realistic phantoms from the test set were compared to unpaired 3D patient data based on noise characteristics and intensity distributions.

Noise magnitude (NM) was measured as the standard deviation of voxel intensities within the liver, a large, mostly homogeneous organ. For the realistic images, liver segmentation was derived from the XCAT/CoMBAT phantom masks, while manual segmentation was adopted for the patient images. Additionally, the noise texture was evaluated using an estimation of the radial noise power spectrum (NPS) [22]. To compare the radial NPS of the realistic and patient images, the Pearson correlation coefficient (NCC) was computed. Also, the similarity of intensity histograms between the two groups was quantified using the Pearson Correlation Coefficient, HistCC.

NM of the liver was computed for each liver volume and averaged across all volumes. For NPS calculation, a region of interest (ROI) of size 64 × 64 × 20 voxels was manually extracted from both realistic and real livers. Finally, the average volumetric histogram was computed for generated and patient groups, and their correlation was measured.

#### Patient-specific phantoms and dosimetric evaluation

Starting from the hold-out patient CTs, patient-specific XCAT phantoms were constructed by segmenting each organ of the patient and assigning it the corresponding CT number from the XCAT simulation. Organs at risk (OARs) were segmented based on the masks from the radiotherapy plan, while the remaining organs were segmented using thresholding and manual refinement. Then, CycleGAN CT was employed to predict the corresponding realistic CT version, which now relied on a proper ground-truth (i.e. original patient CT data). Paired evaluation was therefore performed slice-by-slice between the original patient CT scans and their corresponding CycleGAN-generated realistic CT reconstructions, using the paired metrics outlined in Section 3.4.1. The metrics were computed for every slice of all patients and then averaged to obtain the final results.

A dosimetric analysis was then conducted to assess the realism of the generated CT volumes.

A carbon-ion radiotherapy (CIRT) plan was optimized on the CT scan of one of the testing patients (i.e., P17) using the RayStation Treatment Planning System. OARs, gross tumor volume (GTV), and planning target volume (PTV) were segmented by a radiation oncologist. The relevant OARs included structures such as the aorta, pancreas, duodenum, bowel, and colon. The treatment plan was optimized for both a single-beam and a two-beam setup, with a prescribed clinical dose of 43 Gy and a fractionation scheme of 10 sessions. The treatment based on carbon ions was simulated here as a worst-case scenario, considering that carbon ions are more sensitive than other particles to range variations due to organ motion.

In this setting, a direct dosimetric comparison was performed between the original CIRT plan on the patient CT and the recalculated plan on the corresponding realistic CT phantom. The recalculated plan was evaluated based on dose-volume histogram (DVH) metrics (GTV D_95%,_ i.e., the minimum dose that 95% of the GTV volume receives, PTV D_95%_ and D_2%_ on OARs, i.e., the maximum dose received by the most exposed 2% of an OAR) with respect to the original plan.

#### External validation and generalization assessment

To evaluate the generalizability of the CycleGAN CT network, an external validation was performed using 5 CT scans from The Cancer Imaging Archive (TCIA), selected from multiple diverse datasets. Starting from each patient CT, a patient-specific XCAT phantom was constructed by segmenting relevant organs and assigning appropriate CT numbers derived from the XCAT simulation, as described in Section 3.4.3. In this case, organ segmentations were obtained using TotalSegmentator [47], supplemented by thresholding and manual refinement for bone structures. Subsequently, the trained CycleGAN CT model was applied to generate corresponding realistic CT images from these phantoms.

Quantitative assessment was conducted via paired evaluation between the original patient CT scans and their corresponding CycleGAN-generated realistic CT reconstructions, performed slice-by-slice using the paired metrics outlined in Section 3.4.1. The metrics were computed for every slice of all patients and then averaged to obtain the final results.

### Validating synthetic CT from MRI data

In the context of an MRI-only workflow in CIRT, a conditional GAN was proposed in the work of Parrella et al. [36] to generate synthetic CT starting from MRI data of the abdominal site, as described in Section 2.2. The cGAN was optimized to work on three channels associated with air, bone, and soft tissues, and was trained on thirty-nine MRI-CT volume pairs. Five held-out MRI volumes were then used for the evaluation of the network’s performance according to (i) a CT-based approach where the MRI was divided into the 3 channels through thresholding on the planning CT, and (ii) an MRI-only scenario where the MRI was separated into the three channels by means of manual segmentation. In (i) the sCT was compared to the planning CT, while in (ii) the sCT was compared to a pseudo-CT generated by deforming the original CT on MRI via DIR. In both cases, however, the CT used for similarity evaluation did not constitute the real ground truth. Indeed, in the first case the planning CT suffered from interfractional motion with respect to the synthetic CT, whereas in the second case, the results were affected by DIR uncertainties.

To properly validate the network’s performance and, at the same time, demonstrate the utility and the realism of the generated data, we proposed, for the first time, using the paired set of 55 realistic CT-MRI volumes as a multi-modal ground-truth dataset characterized by a perfect matching of structures on corresponding CT-MRI images. Therefore, realistic phantom MR images were provided as input to the cGAN to predict the corresponding sCT, which was then compared with the realistic CT ground-truth by means of one-to-one similarity metrics, including SSIM, MAE, peak signal-to-noise ratio (PSNR), normalized cross correlation (NCC), and root mean squared error (RMSE).

Realistic MRIs were divided into the three channels through thresholding on the corresponding realistic CT, replicating the CT-based approach described before. However, since in this case the realistic CT and MRI are perfectly matched anatomically, this approach also coincides with the MRI-only scenario.

## Results

### Hyper-parameter tuning

A systematic hyperparameter tuning was conducted to identify the optimal training configuration for both CycleGAN CT and CycleGAN MRI. The tuning process focused on three key parameters: the weighting of the intensity loss (*λ*_int_), the weighting of the gradient difference loss (*λ*_gdl_), and the learning rate of the discriminator (LR_d), while keeping the generator learning rate fixed at 2e−4. A total of six configurations were tested for MRI and five for CT, as reported in Table 2. Quantitative evaluation of each model was then performed using the paired and unpaired metrics.

**Table 2:** Quantitative results from hyper-parameter tuning based on paired and unpaired evaluations

**MRI**				**Paired metrics**						**Unpaired metrics**			
	***λ***_**int**_	***λ***_**gdl**_	**LR_d**	**MAE**	**SSIM**	**FSIM**	**EPR**	**EGR**	**LPIPS**	**NM (patient)**	**NM (phantom)**	**HistCC**	**NCC**
**1st**	0.3	0.3	2e − 4	25.3 ± 0.8	0.37 ± 0.01	0.81 ± 0.04	0.49 ± 0.01	0.94 ± 0.05	0.30 ± 0.03	8.7 ± 1.8	12.8 ± 0.8	0.997 ± 0.002	0.88 ± 0.05
**2nd**	0.3	0.3	2e − 5	21.3 ± 0.8	0.4 ± 0.01	0.86 ± 0.01	0.58 ± 0.01	0.88 ± 0.06	0.27 ± 0.03	8.7 ± 1.8	11.3 ± 0.6	0.997 ± 0.001	0.90 ± 0.05
**3rd**	0.4	0.4	2e − 4	26.2 ± 0.06	0.29 ± 0.02	0.70 ± 0.03	0.28 ± 0.05	**0.99 ± 0.05**	0.28 ± 0.07	8.7 ± 1.8	6.6 ± 0.5	0.997 ± 0.001	**0.94 ± 0.06**
**4th**	0.4	0.4	2e − 5	18.8 ± 1.2	0.41 ± 0.01	0.88 ± 0.04	0.58 ± 0.03	0.81 ± 0.08	**0.23 ± 0.02**	8.7 ± 1.8	9.9 ± 0.3	0.996 ± 0.001	0.89 ± 0.04
**5th**	**0.5**	**0.5**	**2e − 4**	**18.8 ± 0.6**	**0.71 ± 0.04**	**0.88 ± 0.01**	**0.66 ± 0.02**	1.06 ± 0.08	0.25 ± 0.03	8.7 ± 1.8	**9.6 ± 0.7**	**0.998 ± 0.001**	0.90 ± 0.04
**6th**	0.5	0.5	2e − 5	17.6 ± 0.8	0.42 ± 0.01	0.88 ± 0.01	0.6 ± 0.02	0.88 ± 0.02	0.23 ± 0.03	8.7 ± 1.8	10.3 ± 1.6	0.997 ± 0.002	0.88 ± 0.03
**CT**				**Paired metrics**						**Unpaired metrics**			
	***λ***_**int**_	***λ***_**gdl**_	**LR_d**	**MAE**	**SSIM**	**FSIM**	**EPR**	**EGR**	**LPIPS**	**NM (patient)**	**NM (phantom)**	**HistCC**	**NCC**
**1st**	0.5	0.5	2e − 5	123.9 ± 13.4	0.62 ± 0.04	0.76 ± 0.01	0.42 ± 0.03	0.94 ± 0.07	0.26 ± 0.02	21.5 ± 2.6	34.1 ± 3.1	0.96 ± 0.04	0.80 ± 0.06
**2nd**	**1**	**1**	**2e − 5**	**103.8 ± 10.6**	**0.67 ± 0.05**	**0.83 ± 0.01**	**0.48 ± 0.02**	0.83 ± 0.08	0.26 ± 0.3	21.5 ± 2.6	**27.4 ± 2.3**	**0.97 ± 0.04**	0.82 ± 0.08
**3rd**	1.5	1.5	2e − 5	119.9 ± 12.2	0.62 ± 0.04	0.76 ± 0.01	0.43 ± 0.03	**1.01 ± 0.03**	**0.25 ± 0.02**	21.5 ± 2.6	29.5 ± 2.1	0.96 ± 0.04	**0.85 ± 0.05**
**4th**	2	2	2e − 5	105.6 ± 8.3	0.63 ± 0.04	0.79 ± 0.01	0.46 ± 0.03	0.93 ± 0.06	0.26 ± 0.02	21.5 ± 2.6	29.3 ± 3.2	0.94 ± 0.05	0.70 ± 0.05
**5th**	2.5	2.5	2e − 5	105.8 ± 8.5	0.63 ± 0.04	0.80 ± 0.02	0.47 ± 0.03	0.93 ± 0.07	**0.25 ± 0.02**	21.5 ± 2.6	29.2 ± 4.1	0.92 ± 0.06	0.59 ± 0.08

The table summarizes the results obtained by varying the values of key hyperparameters (*Int*, intensity loss weight; *Gdl*, gradient difference loss weight; *LR_d*, discriminator learning rate) for both MRI and CT CycleGAN models. For each configuration, paired metrics were computed between the generated realistic images and their corresponding original phantoms to assess anatomical fidelity. Unpaired metrics were calculated to evaluate the realism of the generated images against real patient data. All results are reported as Average ± St. Dev. and expressed in arbitrary units

For CT, preliminary experiments demonstrated that using a discriminator learning rate of 2e−4 led to unbalanced adversarial training: the discriminator rapidly overpowered the generator, easily distinguishing real from fake samples and preventing the generator from receiving meaningful gradient feedback. To mitigate this issue and promote stable training dynamics, LR_d was fixed at 2e−5 for all configurations tested during CT tuning.

For MRI, the 5th configuration (*λ*_int_ = 0.5, *λ*_gdl_ = 0.5, and LR_d = 2e−4) achieved the best trade-off between structural preservation and realism. For CT, the 2nd configuration (*λ*_int_ = 1, *λ*_gdl_ = 1, and LR_d = 2e−5) yielded the most favorable results. These configurations were therefore selected for the generation of realistic phantoms used in the subsequent analyses.

### Realistic phantoms

**Fig. 4:**
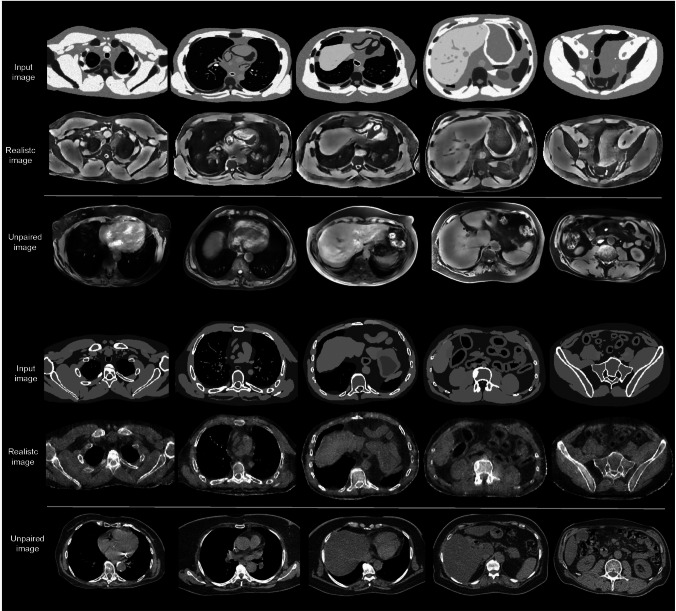
Qualitative representation of realistic phantoms generated using CycleGANs. Examples of axial slices for the test set are reported for both MRI (top) and CT (bottom). For each imaging modality, the original input images from CoMBAT and XCAT are displayed along with their corresponding realistic version produced by CycleGANs. Unmatched patient images are included for qualitative comparison of the results. The slices were extracted from random patients. Each column corresponds to a specific anatomical region: (1st) random slice within the upper body, (2nd) from the lung region, (3rd) at the level of the liver, (4th) from the abdominal area, and (5th) from the pelvic region

Figure 4 shows examples of axial slices from the XCAT/CoMBAT phantoms along with their corresponding realistic versions generated by CycleGANs, as well as patient images for qualitative comparison. The results shown correspond to the best-performing hyperparameter configurations for MRI (5th row) and CT (2nd row), selected based on the quantitative metrics in Table 2. The visual style of the realistic images closely aligned with that of actual patient scans, while preserving the anatomical structure of the original phantoms.

#### Paired evaluation

The FSIM and SSIM metrics demonstrated effective preservation of image structures and features, with the realistic MRI data yielding an FSIM value of 0.88 ± 0.01, compared to 0.83 ± 0.01 for the CT data. The EPR values, approaching 0.5 for the CT dataset and exceeding 0.5 for the MRI dataset, further indicated effective preservation of original organ edges. Additionally, the EGR metric approached 1, suggesting minimal introduction of artificial edges in the synthetic images. The MRI mean LPIPS value of 0.25 ± 0.03 and CT mean LPIPS of 0.26 ± 0.03 indicate a moderate perceptual difference. Since lower LPIPS means higher similarity, values near zero would imply little to no style change, meaning the phantom remains unaltered. Therefore, a moderate LPIPS reflects a balance between preserving anatomy and enabling realistic style transformation.

#### Unpaired evaluation

**Fig. 5:**
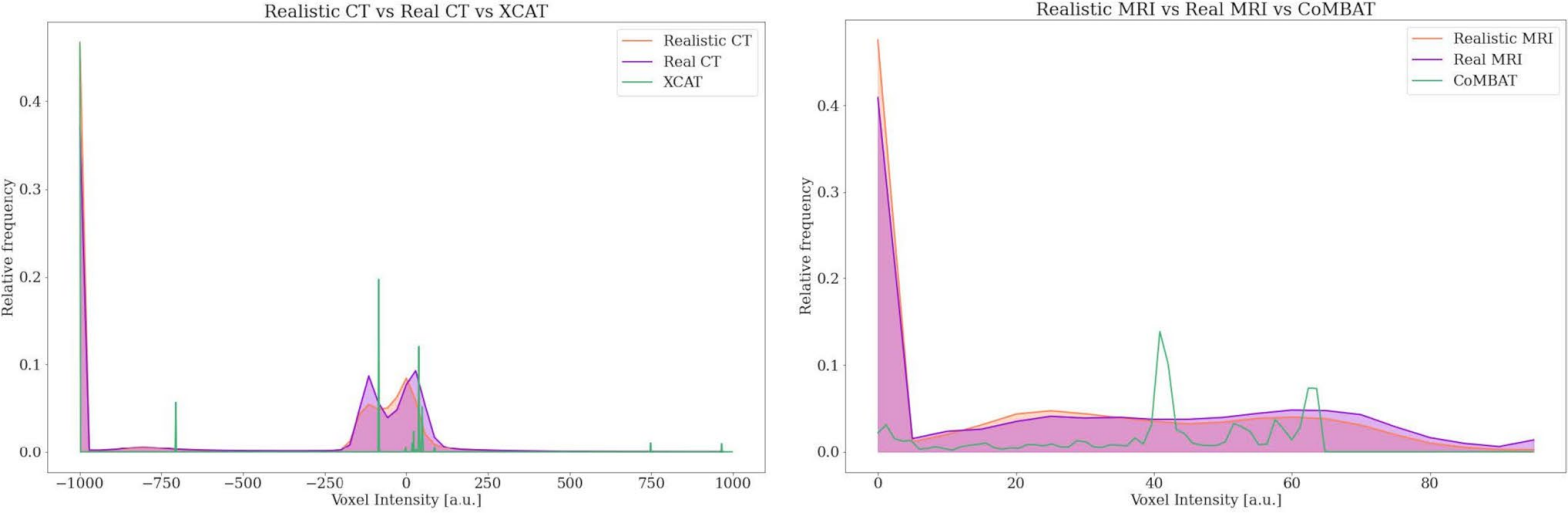
Histogram analysis. (Left) Average intensity histograms of patient CTs, realistic phantom CTs, and original XCAT. (Right) Average intensity histograms of patient MRIs, realistic phantom MRIs, and original CoMBAT

For MRI, the mean NM in the liver of realistic data was 9.6 ± 0.7 a.u., closely matching the 8.7 ± 1.8 a.u. computed from patient data. In contrast, for CT, a higher NM was observed in the realistic phantoms (27.4 ± 2.3 HU) compared to the patient data (21.5 ± 2.6 HU). A high NCC for both modalities (0.90 ± 0.04 for MRI and 0.82 ± 0.08 for CT) suggested that the noise texture was replicated realistically. From a histogram perspective, Fig. 5 illustrates that the average intensity distribution of the realistic images closely matched that of the patient images for both modalities. For CT, the two peaks associated with soft tissues were replicated in the realistic average histogram, as well as the peak relative to lung tissue. This observation was further supported by the HistCC value, which reached 0.99 for MRI and 0.97 for CT.

#### Patient-specific phantoms and dosimetric evaluation

The quantitative results on the hold-out patient set are summarized in Table 3, showing a mean MAE of 86.9 ± 19.9 HU and SSIM of 0.64 ± 0.02, indicating a good agreement between the CycleGAN-generated realistic CTs and the original patient CTs. FSIM and LPIPS further confirmed the quality of the synthetic images.

**Table 3:** Hold-out vs. external validation

	MAE	SSIM	FSIM	EPR	EGR	LPIPS
Hold-out	86.9 ± 19.9	0.64 ± 0.02	0.83 ± 0.01	0.52 ± 0.04	1.04 ± 0.15	0.22 ± 0.02
External validation	103.4 ± 35.2	0.74 ± 0.03	0.87 ± 0.01	0.62 ± 0.04	0.95 ± 0.09	0.16 ± 0.03

Image similarity metrics comparing CycleGAN-generated and original CTs for hold-out and external validation sets. Values are reported as Average ± St. Dev. The metrics were computed for every slice of all patients and then averaged

**Fig. 6:**
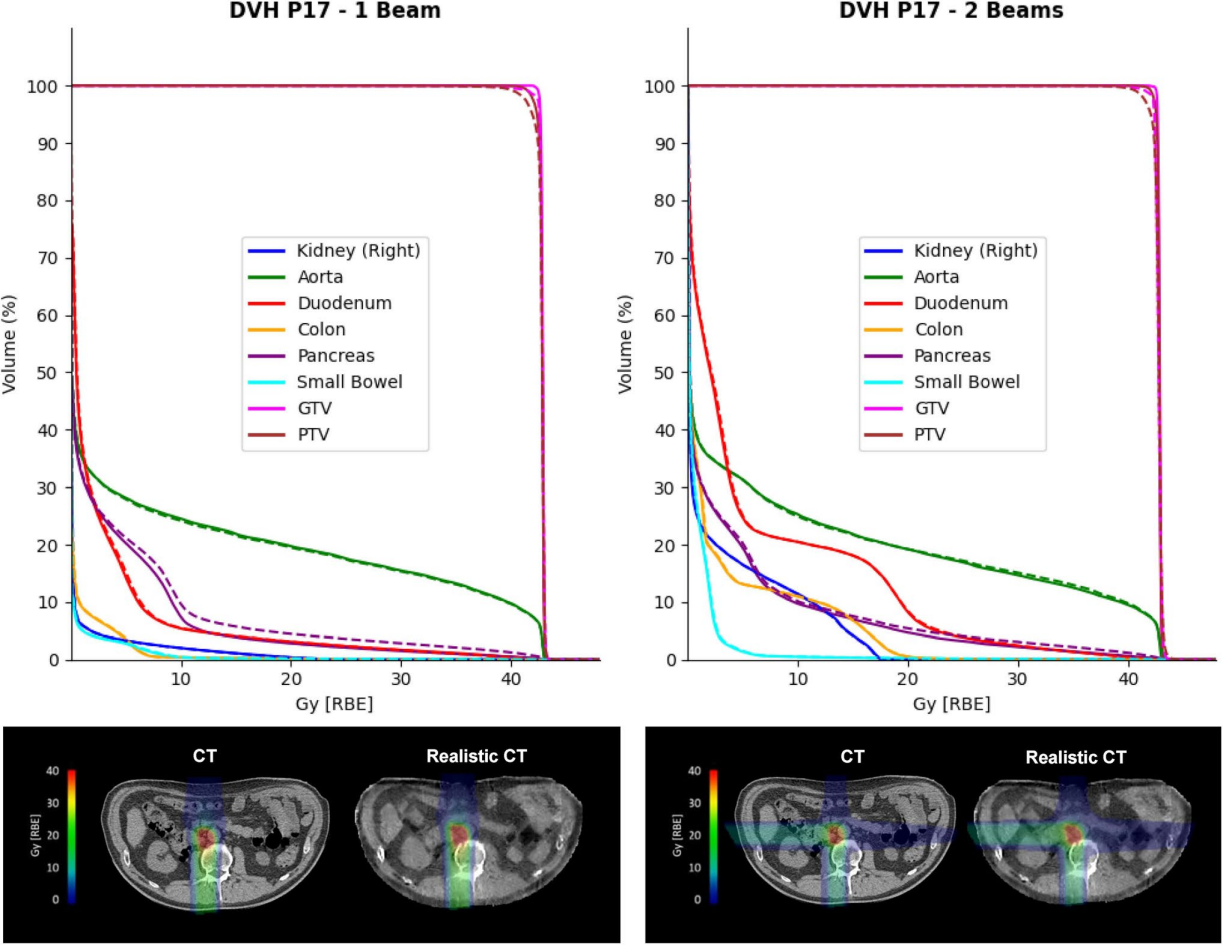
Dosimetric evaluation. (Top) DVH comparison on patient P17; (Bottom) original CIRT plan (RBE) and realistic CT-based recalculation for patient P17. (Left) Results for one-beam configuration and (Right) for two-beam setup

**Fig. 7:**
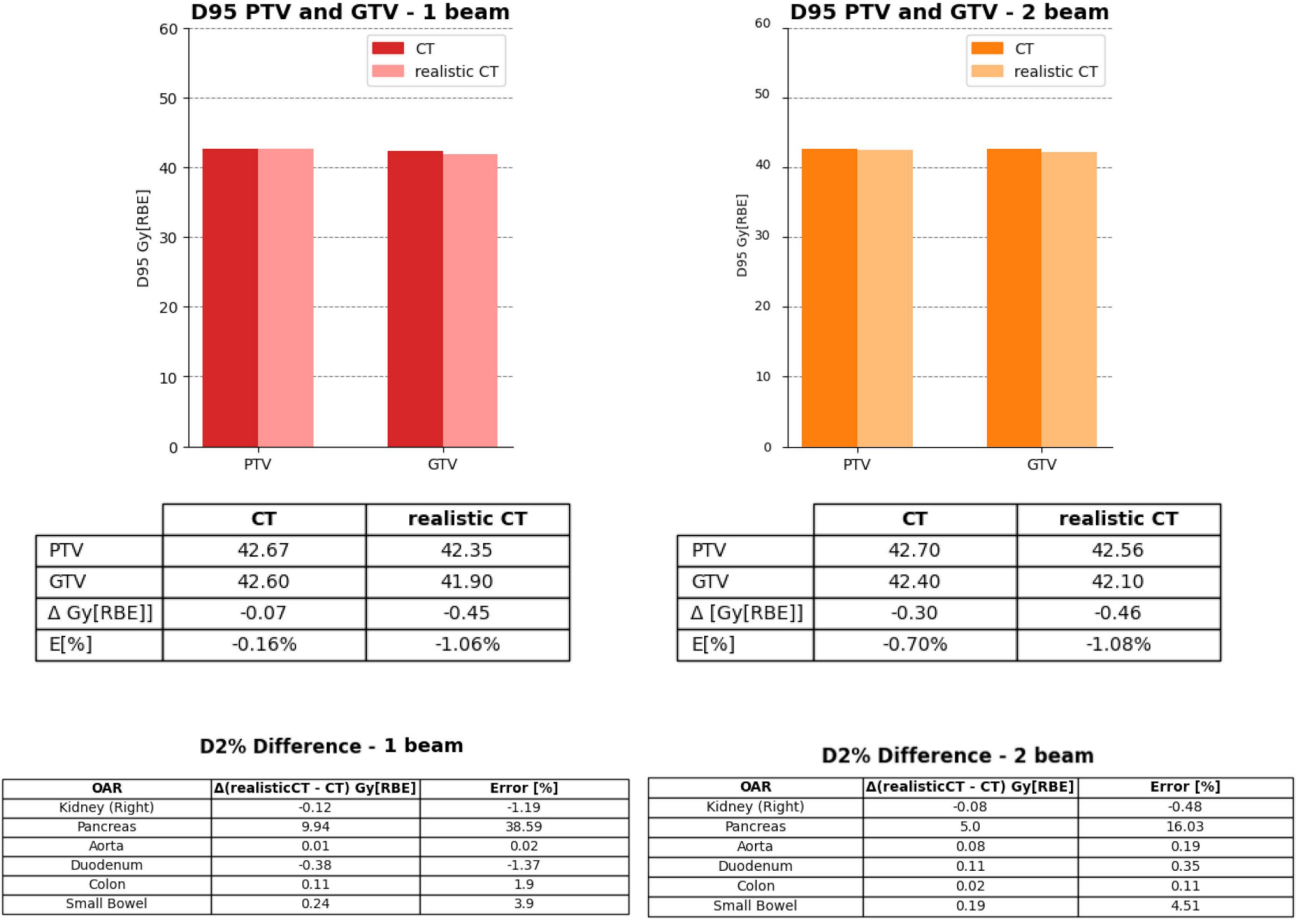
DVH metrics. (Top) D_95%_ values on GTV and PTV in the original plan (CT) and the recalculated one (realistic CT) for P17 for the two-beam configurations. The table contains the dose values and the dose difference Δ (realistic CT–CT) Gy [RBE], along with the error relative to the prescribed dose E [%]. (Bottom) D_2%_ difference (realistic CT–CT) for the main OARs and relative error with respect to the original plan

Figure 6 shows the DVH comparison for patient CT and dose recalculation on the corresponding realistic CT for both the simulated setups (i.e., one or two beams). The GTV and PTV D_95%_, as well as the *D*_2%_ on the OARs, are displayed in Fig. 7, expressed in terms of dose difference Δ (realistic CT–CT) and relative error with respect to the original plan. The recalculated dose distribution for GTV and PTV showed good reproducibility with respect to the prescribed dose, with a maximum error on GTV of − 0.45 Gy [RBE] and − 0.46 Gy [RBE] for the one-beam and two-beam configurations, corresponding to relative errors of − 1.06% and 1.08%, respectively.

The *D*_2%_ relative errors on OARs were below 4% in absolute terms, although the pancreas reached a peak relative error of 38.59% (9.94 Gy [RBE]) for the single-beam study and an error of 16.03% (5.0 Gy [RBE]) for the double-beam study.

#### External validation

For the external validation dataset from TCIA, the results in Table 3 indicate a slightly higher MAE of 103.4 ± 35.2 HU but improved structural similarity (SSIM 0.74 ± 0.03) and FSIM (0.87 ± 0.01) compared to the hold-out set. This suggests overall image quality and fidelity when comparing the synthetic CTs with the real patients’ data. The perceptual metric LPIPS was 0.16 ± 0.03, supporting enhanced visual similarity.

#### Computational cost

Each training epoch of the CycleGAN model required approximately 2.17 min to process the data. The inference speed for generating a realistic XCAT volume, which involved dividing each 2D slice (around 200 slices per volume) into patches, performing predictions on each 256 × 256 pixel patch, and subsequently fusing the patches, took around 10 s. The training process was conducted on an NVIDIA QUADRO P5000 GPU with 16 GB of RAM, leveraging TensorFlow 2.11.0 and Python 3.7 for implementation.

### Validation of the literature cGAN generating synthetic CT from MRI

The results in Table 4 present a comparison between the predicted sCT generated from MRI, validated using the proposed realistic phantom data (first row), and those reported by Parrella et al. [36] on test patient data using both the CT-based approach (second row) and the MRI-only scenario (third row). The similarity metrics suggest a phantom-based validation well aligned with patient test data from the CT-based approach, confirming a strong correlation between patient and realistic data. Particularly, the phantom-based results fell between the overly optimistic outcomes of the CT-based approach and the suboptimal results of the MRI-only simulation. Indeed, examining the MAE across different tissue types (air, bone, and soft tissue), the results on phantoms are higher than the CT-based ones, particularly in the air channel (73.2 ± 11.3 HU compared to 54.4 ± 11.5 HU). At the same time, our results were significantly better with respect to the MRI-only case. Indeed, the evaluation in terms of NCC (0.94 ± 0.02), PSNR (27.7 ± 0.4), and RMSE (99.3 ± 5.8 HU) demonstrated an accurate reproduction of the synthetic volumes with respect to a simulated MRI-only case (with NCC = 0.8 ± 0.1, PSNR = 21 ± 1.5, and RMSE = 181.1 ± 11.9 HU).

**Table 4:** Validation of sCT from MRI

	MAE air [H.U.]	MAE bone [H.U.]	MAE soft [H.U.]	MAE tot [H.U.]	RMSE [H.U.]	SSIM	PSNR [dB]	NCC
**Realistic ph. validation**	73.2 ± 11.3	85.4 ± 6.0	62.5 ± 5.2	64.6 ± 5.5	99.3 ± 5.8	0.62 ± 0.02	27.7 ± 0.4	0.94 ± 0.02
Patient test (CT-based)	54.4 ± 11.5	86 ± 10.8	55.4 ± 3.4	57.1 ± 2.8	99.7 ± 4.9	0.67 ± 0.06	27.6 ± 0.7	0.91 ± 0.03
Patient test (MRI-only)	279 ± 142.5	154.9 ± 22.9	75 ± 8.1	88.2 ± 9.9	181.1 ± 11.9	0.59 ± 0.08	21 ± 1.5	0.8 ± 0.1

(Realistic phantom validation) similarity metrics between sCT and the realistic CT of ground-truth, (Patient test CT-based) similarity metrics between the sCT and the reference (i.e., planning) CT affected by MRI-CT inter-acquisition organ motion, (Patient test MRI-only) similarity metrics between the sCT and the CT deformed on MRI through DIR affected by registration uncertainties. Metrics are reported as Average ± St. Dev

## Discussion

In this study, we generated realistic patient-like CT-MRI volumes using CycleGANs, starting from the XCAT and its MRI version, to provide reliable ground truth data for the validation of deep learning-based cross-modality synthesis techniques in radiotherapy. For each image modality, the network structure, composed of two GANs, successfully learned the phantom-to-patient data mapping, relying on unpaired patches from the phantom and patient domains. To demonstrate the quality and the realism of the generated realistic phantoms, we conducted multiple evaluations, including a dosimetric comparison between a patient CT-based plan and a realistic phantom CT-based recalculation. In addition, we performed external validation on unseen patient data from TCIA to assess the generalizability of the trained CycleGAN CT model beyond the training cohort. Finally, we assessed the quality of the generated data by investigating their ability to replace patient CT-MRI data in the validation of a literature-based network that synthesizes CT images from MRI scans for application in CIRT of the abdominal site.

CycleGAN MRI and CycleGAN CT were optimized through hyperparameter tuning, allowing us to balance the trade-off between anatomical fidelity and visual realism. Specifically, high weights on intensity and gradient difference losses helped preserve the structural features of the original phantoms but resulted in less realistic textures. Conversely, lower weights led to more patient-like appearances at the cost of some morphological deviation from the ground truth. The final settings were chosen to ensure realistic output while maintaining sufficient structural consistency for use in validation tasks.

The resulting phantoms closely resembled patient data, with realistic noise texture added to the originally noise-free phantom. The quantitative metrics from paired evaluation confirmed a good preservation of the original morphological information, ensuring structural alignment between corresponding CT/MRI phantom images. This structural fidelity was crucial, as it ensured that realistic datasets could be used as ground truth for validation purposes. From the unpaired metrics, the histogram analysis showed that the intensity profiles of the realistic data closely mirrored those of the patient data, with HistCC metric reaching 0.97 for CT and 0.99 for MRI datasets. Additionally, we validated the generated phantoms based on their dosimetric accuracy. The recalculation of dose distributions on realistic CT images demonstrated good agreement with the dose plans based on the patient CT. While overall agreement was strong, discrepancies were observed for certain OARs, notably the pancreas, which is adjacent to high-dose regions. These discrepancies are mainly due to anatomical complexities and dose gradients associated with the simulation of a treatment based on heavy ions (i.e., worst-case scenario).

Given the small training dataset, we assessed the generalizability of CycleGAN CT on unseen data from TCIA and compared the results with the hold-out dataset. This evaluation was based on patient-specific XCAT phantoms created from segmented hold-out and public patient CTs, allowing for slice-by-slice paired comparison between original and CycleGAN-generated CTs using quantitative similarity metrics. External validation showed slightly better similarity metrics, likely due to anatomical differences (i.e., patients with smaller body areas have more background regions in their images, thus inflating similarity scores). Nonetheless, the quality of the resulting realistic images remained consistent among external CTs and hold-out cases, thus demonstrating the generalizability of the network. Patch-wise training was key in offering greater sampling diversity, reducing the risk of overfitting and contributing to the model’s ability to perform well on unseen data.

We subsequently proposed using these paired realistic phantoms as an augmented test-bed to validate a deep-learning network previously developed by Parrella et al. [36] for generating synthetic CT from MRI of the abdomen. The advantage of using realistic data here was the perfect anatomical correspondence between the CT and MRI, something difficult to achieve in patients due to motion or alignment errors between different imaging modalities. This anatomical correspondence, along with its numerosity (i.e., 55 CT-MRI volume pairs), enabled a more accurate evaluation of the deep-learning network’s performance, which had previously been tested on a limited number of unpaired data (i.e., 5 patient volumes without a ground truth).

The phantom-based validation produced results consistent with those from patient data in the CT-based approach from Parrella et al. [36], showing that the phantoms can effectively be a substitute for patient datasets in this type of validation. Moreover, the fact that the results fell between the patient CT-based and the patient MRI-only scenarios further confirms the feasibility of using such synthetic datasets for the purpose of deep-learning network validation. This is because the results from the CT-based were overly optimistic, since MRI was divided into the three channels based on CT-derived masks, so that the synthetic CT replicated the air pockets and the bone structures of the reference CT. On the other hand, in the MRI-only simulation, the results were highly affected by the accuracy of DIR, which was not able to fully compensate for different air cavities and inter-acquisition motion. Our phantom-based approach, which used fully aligned CT-MRI volumes, eliminated these issues.

By providing a reliable, anatomically consistent, and noise-realistic dataset, these phantoms could serve as a robust test-bed for validating new deep learning-based cross-synthesis techniques without the variability and ethical concerns associated with patient data. Moreover, the generated phantoms allow for the creation of an augmented test dataset that is numerically robust, thus enabling a more comprehensive evaluation of model performance. Although the synthetic phantoms were representative of the specific scanner protocol for which the network validation was intended, the approach is flexible, and the network could be easily retrained with patient datasets that reflect the imaging characteristics required for future applications. Indeed, the proposed method could be easily extended to other anatomical sites and image modalities (e.g., cone beam CT or other MRI weightings).

Despite the promising results, several limitations remained. First, the anatomical variability of the generated phantoms was limited by the XCAT framework, which may not fully capture the diversity of anatomical and pathological conditions encountered in clinical practice. Future work could address this by integrating advanced generative models capable of introducing variability in organ shapes, sizes, and internal textures, possibly using statistical shape models [48], or even combining multiple phantom sources to increase diversity.

Additionally, introducing lesions and pathologies into the phantoms would make the dataset more suitable for tasks related to disease detection and treatment planning, broadening its applicability to pathology-specific applications. However, for our application, we do not expect the performance of the CycleGAN to be significantly affected by the absence of pathology, as the model learns domain-level texture and intensity mappings without explicitly interpreting or relying on specific anatomical or pathological content. Moreover, adopting self-supervised learning strategies may help models better capture subtle anatomical and textural features without relying on large, annotated datasets [49]. Similarly, multimodal fusion approaches, integrating information from both CT and MRI data during training, could further improve the realism and cross-modality consistency of the generated phantoms, thus enhancing their generalizability across different scanners and acquisition protocols [50].

Future work could also explore using these realistic phantoms for data augmentation during the training of neural networks, potentially mitigating the limited availability of large-scale clinical datasets and improving model generalization.

While GANs currently represent the most widely adopted solution for realistic phantom generation, emerging generative approaches—such as diffusion models and Transformer-based architectures—have shown superior image quality in recent studies compared to GANs [51]. Diffusion models learn data distributions through a progressive denoising process [52], whereas Transformers leverage self-attention mechanisms to capture long-range dependencies and enhance structural consistency [53]. However, these methods come with significant computational overhead: diffusion models require iterative sampling steps, and Transformers involve resource-intensive self-attention calculations [54]. These factors may limit their current practicality for large-scale phantom library creation, although future studies should be focused on comparing these techniques to evaluate the optimal strategy for the generation of accurate realistic computational phantoms.

## Conclusions

In this study, we demonstrated that realistic phantoms generated through a CycleGAN-based framework were highly beneficial for medical image analysis, particularly in the context of deep-learning model validation where paired multimodal data were limited or unavailable. The realistic data closely replicated the appearance and the dosimetric properties of patient data and preserved the original anatomy provided by computational phantoms. Therefore, the generated phantom data offered a two-fold advantage: they served as a reliable ground truth for validation and extended the test dataset, providing a more robust and comprehensive evaluation of deep-learning models in medical imaging. Specifically, we used these realistic synthetic CT-MRI volumes to validate a deep-learning model that synthesized CT images from MRI scans for abdominal carbon ion radiotherapy. Our results showed that the use of these realistic data provided consistent and reliable performance metrics when compared to patient-based validation, reinforcing the potential of realistic phantom data as a substitute for patient data in model evaluation.

From an innovation perspective, our method addressed a critical challenge in synthetic CT generation by providing a controlled yet realistic dataset that enabled quantitative validation while avoiding the ethical and statistical difficulties associated with clinical data acquisition. For CT, we demonstrated good generalizability through external validation; however, for MRI, the generalizability of the method to different scanners, imaging settings, or anatomical regions remains to be explored. Future work will focus on expanding the variability of synthetic datasets, incorporating pathological conditions, and further validating the approach across multiple imaging protocols and clinical scenarios.

## Supplementary Information

Below is the link to the electronic supplementary material.

Supplementary file1 (DOCX 259 KB)

## Data Availability

The code developed by the authors for this study is available at: https://github.com/camagnif/Realistic-MRI-CT-XCAT-Phantoms-for-Validating-MRI-Based-Deep-Learning-SynthCT-Methods.
