# Supplementary material for: Generation of multimodal realistic computational phantoms as a test-bed for validating deep learning-based cross-modality synthesis techniques
Source: Med Biol Eng Comput. 2025 Sep 27;64(1):263–84. doi: 10.1007/s11517-025-03437-4 (PMC12868042; doi:10.1007/s11517-025-03437-4)
Supplement: Supplementary file 1 — Supplementary file1 (DOCX 259 KB) [file 11517_2025_3437_MOESM1_ESM.docx]

Supplementary Material

**S1 Initial approach to validation**

The initial validation approach for a literature-based 3-channel conditional GAN (cGAN) [36], designed to generate synthetic CT (sCT) images from abdominal MRI for radiotherapy applications, utilized original CT/MRI phantoms. Specifically, the CT XCAT phantoms [12] and their corresponding MRI versions, generated using the CoMBAT framework [20], served as ground truth data for validation purposes.

Prior to testing the cGAN on phantom data, the MRI phantoms underwent preprocessing to match the format of the 3-channel cGAN training input. This included denoising, contrast enhancement, background removal, and histogram matching. Bias field correction was deliberately excluded, as synthetic MRIs do not exhibit magnetic field inhomogeneities. The cGAN then generated sCT images, which were compared against the original XCAT CT phantoms serving as ground truth. However, the results showed a notable discrepancy from those obtained using real patient data (see Figure S1). In particular, the Mean Absolute Error (MAE) in bone regions was significantly higher (549.95 HU), primarily due to the limited intensity range of bone structures in the XCAT CT phantoms, which feature only two discrete values. This contrasts with the broader and more continuous intensity distribution present in actual patient data. As illustrated in Figure S1, the sCT histogram exhibits a continuous intensity distribution, whereas the XCAT CT histogram retains a discretized profile with only a few distinct values. This observation is further supported by the quantitative analysis in Table S1. For methodological and metric details, refer to Section 3 of the manuscript.


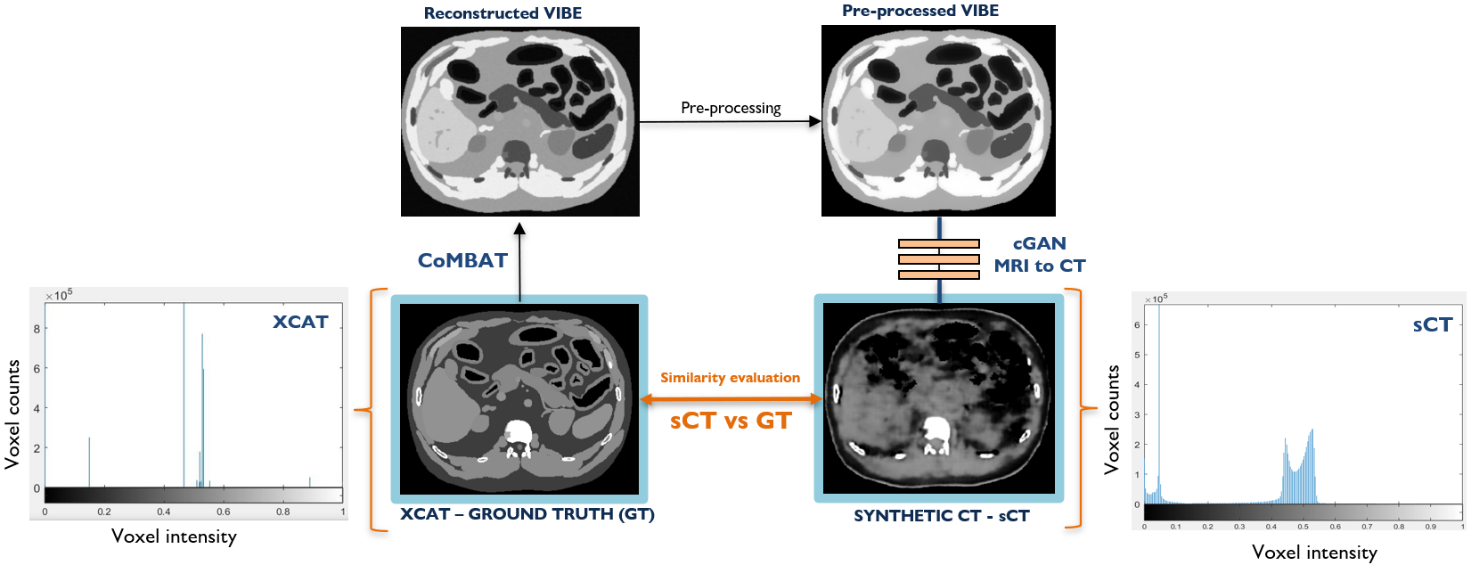
These findings highlight the importance of aligning the test data’s intensity distribution with that of the training data for valid evaluation. Consequently, to preserve the ground truth benefits of using phantoms while ensuring meaningful performance assessment, it became necessary to enhance the phantoms with more realistic imaging characteristics.

**Figure S1** – initial approach to validation.

|  | **MAE tot** | **MAE bone** | **MAE air** | **MAE soft [H.U.]** | **RMSE [H.U.]** | **SSIM** | **PSNR [dB]** | **NCC** |
| --- | --- | --- | --- | --- | --- | --- | --- | --- |
| Results | 110.20 | 549.95 | 279.49 | 88.60 | 179.63 | 0.68 | 24.78 | 0.78 |
| Testing | 57.08 | 86.03 | 54.42 | 55.39 | 99.69 | 0.67 | 27.64 | 0.92 |

**Table S1** – Similarity metrics from initial approach to validation. The quantitative metrics reported in this Table are describe in detail in Section 3.4 of the manuscript.
